# A Robotized Raspberry-Based System for Pothole 3D Reconstruction and Mapping

**DOI:** 10.3390/s23135860

**Published:** 2023-06-24

**Authors:** Salvatore Bruno, Giuseppe Loprencipe, Paola Di Mascio, Giuseppe Cantisani, Nicola Fiore, Carlo Polidori, Antonio D’Andrea, Laura Moretti

**Affiliations:** 1 Department of Civil, Constructional and Environmental Engineering, Sapienza University of Rome, Via Eudossiana 18, 00184 Rome, Italy; salvatore.bruno@uniroma1.it (S.B.); giuseppe.loprencipe@uniroma1.it (G.L.); paola.dimascio@uniroma1.it (P.D.M.); giuseppe.cantisani@uniroma1.it (G.C.); nicola.fiore@uniroma1.it (N.F.); antonio.dandrea@uniroma1.it (A.D.); 2 AIPSS Associazione Italiana Professionisti Sicurezza Stradale, Piazza del Teatro di Pompeo 2, 00186 Rome, Italy; c.polidori@aipss.it

**Keywords:** road surface monitoring and maintenance, pavement potholes, urban road networks, safety at work, low-cost sensors, Raspberry Pi, autonomous robot, digital photogrammetry processing software, GIS technologies

## Abstract

Repairing potholes is a task for municipalities to prevent serious road user injuries and vehicle damage. This study presents a low-cost, high-performance pothole monitoring system to maintain urban roads. The authors developed a methodology based on photogrammetry techniques to predict the pothole’s shape and volume. A collection of overlapping 2D images shot by a Raspberry Pi Camera Module 3 connected to a Raspberry Pi 4 Model B has been used to create a pothole 3D model. The Raspberry-based configuration has been mounted on an autonomous and remote-controlled robot (developed in the InfraROB European project) to reduce workers’ exposure to live traffic in survey activities and automate the process. The outputs of photogrammetry processing software have been validated through laboratory tests set as ground truth; the trial has been conducted on a tile made of asphalt mixture, reproducing a real pothole. Global Positioning System (GPS) and Geographical Information System (GIS) technologies allowed visualising potholes on a map with information about their centre, volume, backfill material, and an associated image. Ten on-site tests validated that the system works in an uncontrolled environment and not only in the laboratory. The results showed that the system is a valuable tool for monitoring road potholes taking into account construction workers’ and road users’ health and safety.

## 1. Introduction

The detection and analysis of road surface defects impact the definition of the most appropriate maintenance strategy to ensure traffic safety and ride comfort [1]. Effective maintenance planning also reduces maintenance costs for road managers who face increasing budgetary constraints [2]. Pavement Management Systems (PMSs) are a systematic methodology to assist decision-makers in identifying the best strategy [3]. In this regard, pavement data collection plays a pivotal role and is the most expensive and time-consuming PMS component [4,5].

The detection and assessment of road surface distress are grouped for flexible pavements into cracking, distortion, disintegration, and skid hazards [6]. Disintegration includes potholes which are local bowl-shaped depressions (usually less than 750 mm diameter) classified into three severity levels—Low (L), Medium (M), and High (H)—based on the pothole diameter and depth according to Table 1 [7].

The progressive pavement disintegration into small parts lost with time and under traffic causes potholes. Freeze-thaw cycles are a common factor in pothole forming, where water seeps into pavement cracks and then freezes, causing the pavement to crack and break apart [8]. The repeated passage of traffic-induced loads, poor construction, and the characteristics of the subgrade soil can contribute to pothole occurrence [9]. In the case of Rome, where the climate is relatively mild, the primary causes of potholes may differ from areas with harsher climates: fatigue and pumping from rain can be significant contributors to pothole formation in areas with heavy traffic and poor drainage. It can cause the road surface to weaken, crack, and eventually break apart, forming potholes. Potholes are frequent in urban areas and imply a significant danger to road users, specifically those with two-wheeled vehicles, and potential damage to vehicles [10]. In 2018 potholes and other pavement obstacles on Italian roads caused the death of 75 motorcyclists and injured another 1773. These numbers represent 8.9% and 3.9% of deaths and injuries among two-wheelers, respectively [11].

Hence, pothole detection during routine inspections and its repair [12] are a crucial challenge for road managers because they limit maintenance costs and extend the pavement service life [13]. On the other hand, promptly repairing potholes can return a quality similar to the original pavement, although patching is considered a pavement defect [14].

The traditional manual visual inspections have been the most commonly used road pothole detection method, but it is time-consuming and expensive, exposing workers to traffic hazards too [15]. Visual inspections of potholes are part of a comprehensive pavement assessment to calculate the Pavement Condition Index (PCI) by counting the number of potholes that are low-, medium-, and high-severity [7]. In recent years automated pothole-detection and measurement methods are becoming increasingly popular as they offer a faster and more efficient way to detect and measure potholes [16]. Based on the latest literature, commonly these methods are classified according to [17]:

- Vision-based methods are fed input visuals (image or video) and include image processing and deep learning algorithms to determine the presence of potholes [18,19,20]. These methods are limited in measuring potholes’ geometric properties, such as depth and volume, because they use two-dimensional (2D) information. In other words, a 2D image or video can only provide us with a two-dimensional representation of a pothole, which makes it difficult to determine its shape and size;
- Vibration-based methods use data collected from inertial sensors inside test vehicles and assume potholes as a significant acceleration impulse [21,22]. However, these methods cannot detect road surfaces (only wheel paths) [23] and the proper identification of abnormal vibration causes (e.g., distinguishing between potholes and pavement bumps) [24];
- Three-dimensional (3D) reconstruction-based methods generate pothole 3D representations, from multiple images of the same scene, in the form of point cloud models, mesh models, and geometric models. By using stereovision-based methods—including photogrammetry and Structure-from-Motion (SfM) techniques—it is feasible to assess the pothole size, shape, and depth and measure its volume accurately. Some commonly used 3D pothole reconstruction-based methods include LiDAR (Light Detection and Ranging) scanning [25], stereo vision [26], and photogrammetry [27]. Various techniques are employed to extract depth and geometric information from images; by identifying corresponding points or features in multiple images, the camersas’ position and orientation is determined, and the 3D structure of the scene can be reconstructed [28]. The quality and accuracy of the 3D model depend on factors such as the number and distribution of images, image resolution, camera calibration, and the effectiveness of feature-matching algorithms [29].
- The photogrammetry approach has been successfully tested by Tion et al. [30] to classify pothole samples in terms of severity levels. In recent years, Atencio et al. [31] developed a method for pothole measurement from 3D models generated from photographs shot by unmanned aerial vehicles (UAVs), determining the optimal flight parameters.

Integrating automated pothole-detection and measurement methods with a Geographic Information System (GIS) can further enhance the efficiency and effectiveness of pavement maintenance and repair efforts by enabling real-time monitoring and analysis of pothole data and facilitating targeted repair and maintenance activities based on accurate and up-to-date information. Once road distresses are detected, their location and characteristics can be captured and georeferenced using GIS, creating a detailed map of pavement failure distribution. Fendi et al. [32] proposed an easy-to-use and cost-effective system for creating a GIS database of geo-tagged photos to help local authorities automate the process of recording and reporting pavement distresses. Nautiyal and Sharma [33] used GIS to store, display, and analyse relevant road data. They developed a tool within GIS to automatically select the most appropriate maintenance technique for each road based on the information stored in GIS. Obaidat et al. [34] used GIS software to analyse data from state-of-the-art technology in pavement management based on the Pavement Condition Index (PCI).

This study aims to overcome the weakness of the pothole 3D reconstruction methodology, which has been revealed in the literature to be the most expensive among the automated methods [35]. The Raspberry Pi technology has been exploited to develop a low-cost prototype for photogrammetric data acquisition. A camera module has been connected to a Raspberry Pi board to capture and save digital images of each pothole from different points of view lined up along predefined circular paths. The latest skill has been made possible by mounting the Raspberry-based prototype on an autonomous and remote-controlled robot to reduce workers’ exposure to live traffic. During field surveys, it works without traffic, protected by a service vehicle. Finally, collected 2D digital photographs have been post-processed using photogrammetry software commonly used in engineering to generate 3D models of objects [36,37]. Significant attention has been paid to the computation of the pothole volume. Data processing is not performed in real-time because the challenge and feature of the system is to be part of a PMS. Indeed, data post-processing should identify the best intervention strategy, and it can require the expertise of multiple technical personnel before filling potholes or scheduling other works. This specific point of the research is a task of the ongoing European research project (InfraROB) focused on a 3D printer able to extrude a bitumen-based mixture for filling in potholes. The pothole volume computation aims to evaluate the amount of material needed to fill in the pothole and to be uploaded on the small tank of the 3D printer. The photogrammetry processing software outputs have been checked by comparison with laboratory tests on a tile made of asphalt mixture, reproducing a real pothole. Integrating a Global Positioning System (GPS) module and GIS technologies allowed the identification of potholes on a map. Each pothole is represented by a point on the map, with information about its centre, volume, backfill material, and an associated image.

On-site tests have been performed considering 10 potholes in a private parking area to demonstrate the system works in an uncontrolled environment.

### The InfraROB Project

InfraROB is a project funded by the EU research program Horizon 2020 with the Grant Agreement N. 955337 to maintain road infrastructure integrity, performance, and safety through autonomous robotised solutions and modularisation. It has to be noticed that the European Union’s Horizon 2020 program is funding also the HERON project to develop an integrated automated system and an autonomous ground robotic vehicle for the maintenance of road infrastructures [38].

A specific task of the InfraROB project focuses on a 3D printer to extrude a mixture to repair potholes. A cold asphalt mixture composed of 100% RAP and an innovative bitumen rejuvenator has been designed in InfraROB to repair potholes [39]. The printer is on a small autonomous carrier (TinyMobileRobots [40], TMR) provided by a project partner. The integrated system allows a preventative approach to road maintenance focused on the early stages of road degradation when the potholes are still small. The machine can autonomously reach the position of the pothole to repair while the operator remains parked in a safe roadside area monitoring remotely the robot only, thus reducing workers’ exposure to live traffic. Due to the reduced dimensions of the autonomous carrier and the limited space for the printer allocation, the choice of the mixture had to comply with different constraints. The authors studied a material was studied that did not need compaction to avoid adding a piece of compaction equipment and lighten the carrier. Therefore, the study about the suitable mixture focused on a material that, immediately after the laying, can be compacted by the wheels of the repairing vehicle itself and later by the traffic vehicles. The mixture was studied to achieve and balance conflicting performances of consistency, flowability homogeneity, and internal structure and to be used to repair potholes without preliminary operations (e.g., cutting and cleaning of the asphalt). The final choice was a mixture composed of 100% Reclaimed Asphalt Pavement (RAP) mixed with a rejuvenator [39]: it provides a “green solution” using 100% RAP, that is, without the need to use natural resources.

The dimension of the equipment also limits the maximum weight the small robot can carry. Indeed, the fully loaded weight of the autonomous carrier is 50 kg, and the maximum weight of the mixture that can be loaded is 5 kg since the 3D printer alone weighs 20 kg. Therefore, it could fill in only small potholes: the maximum pothole dimensions to fill-in in a single step are 20 cm-diameter and 2 cm-depth. With these pothole dimensions, the robot can fill 3 potholes per mission. After filling, the autonomous system can return to its base (a van parked in a safe zone) to be re-filled in.

## 2. The Proposed Integrated System

In this section, the authors describe the developed pothole monitoring system. A novel Raspberry-based system is designed to automatically collect a series of 2D images of the potholes, reproduce precise 3D reality models, and measure the properties of the potholes through digital photogrammetry processing software. The proposed monitoring system can survey up to 75 cm-diameter potholes.

**Figure 1:**
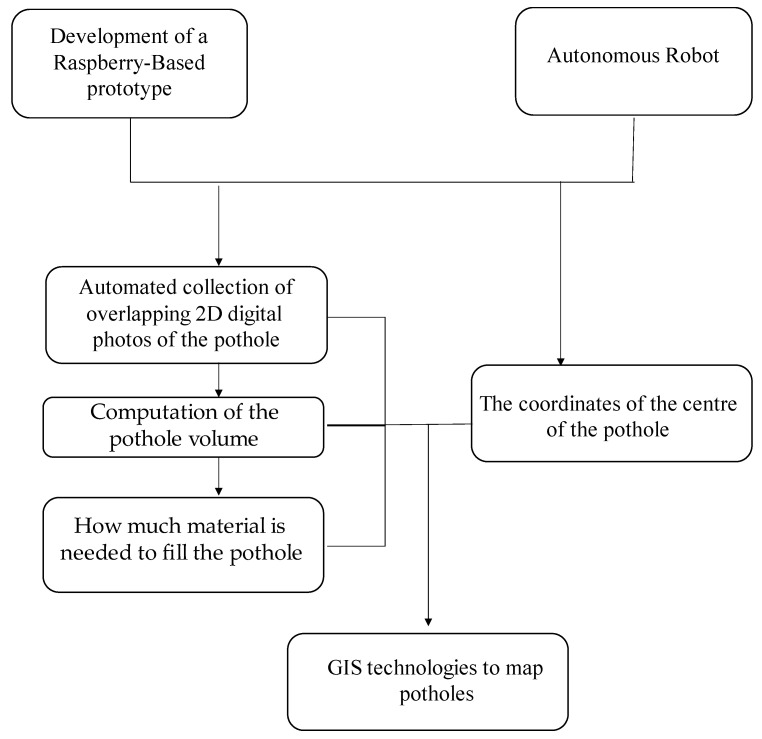
The framework of the overall procedure.

The prototype is integrated into a remote-controlled robotised carrier to capture photos by following a circular path around the potholes, ensuring that each photo overlaps the previous one. A GPS module connected to the Raspberry Pi acquires location data that, combined with GIS technologies, makes it possible to display each pothole on a map as a point, with details about the centre’s location, volume, amount of filling material, and a photo. Figure 1 summarises the overall procedure adopted in this research to reconstruct and map potholes.

The proposed system is currently under development, but it cannot be used in adverse weather conditions both for reasons related to the performance of the fill material (presence of water on the pavement surface) and to avoid potholes during the survey being filled by water or hail that causes variations in the volume calculation. In addition, lighting conditions affect and, in some cases, limit the operation of the system: at the moment, night-time surveys are not possible although they are suitable for safety operational reasons.

### 2.1. Hardware Platform

The Raspberry Pi technology has become popular due to its affordability, versatility, and easy-to-operate [41]. Several applications in road engineering are available in the scientific literature. Some researchers indirectly monitor the road conditions inside vehicles by using a low-cost inertial measurement unit (IMU) and a GPS module connected to a Raspberry Pi Zero W [42,43,44]. Ambrož et al. developed and tested a low-cost device based on the Raspberry Pi single-board computer for assessing road surface friction [45]. Kulkarni and Baligar propose a system that uses a camera module connected to a Raspberry Pi to detect, track, and count vehicles in real-time [46]. Wu et al. introduced a method that combines UAVs, high-resolution cameras, infrared thermography, Raspberry Pi computers, and deep neural networks to provide a cost-effective and automated solution for infrastructure condition assessment [47].

**Figure 2:**
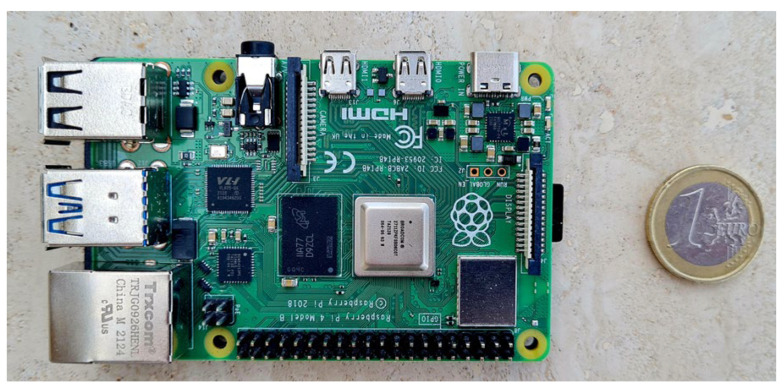
RPI 4B.

In this study, the hardware platform assembled and set up consists of consumer-grade components integrated around a Raspberry Pi 4 Model B 8 GB RAM [48] (Rpi 4B, Figure 2), for a final cost of about €250.00 also with the opportunity to purchase on the most popular e-commerce platforms. The hardware does not have enough computing resources to calculate potholes’ volume using photogrammetric techniques. On the other hand, Raspberry Pi technologies have been exploited to acquire data cost-effectively to support road managers in monitoring activities.

The dimensions of this computer (88 × 58 × 19.5 mm) are like those of a credit card: its compact size makes the device portable. A 3A at 5V power bank powers the Rpi 4B. The Pi 4 has dual-band 802.11ac wireless networking, Bluetooth 5.0, Gigabit Ethernet, 2 USB 3.0 and 2 USB 2.0 ports. It also has dual micro-HDMI ports. The operating system installed on the prototype developed in this study is Raspberry Pi OS, based on the Debian Linux distribution. One feature of the Raspberry Pi 4 board is the General-Purpose Input/Output (GPIO) header with 40 pins to connect different sensors. Additionally, the camera port between the HDMI and Ethernet port allows easy connection of official Raspberry Pi cameras.

#### 2.1.1. GPS Module

**Figure 3:**
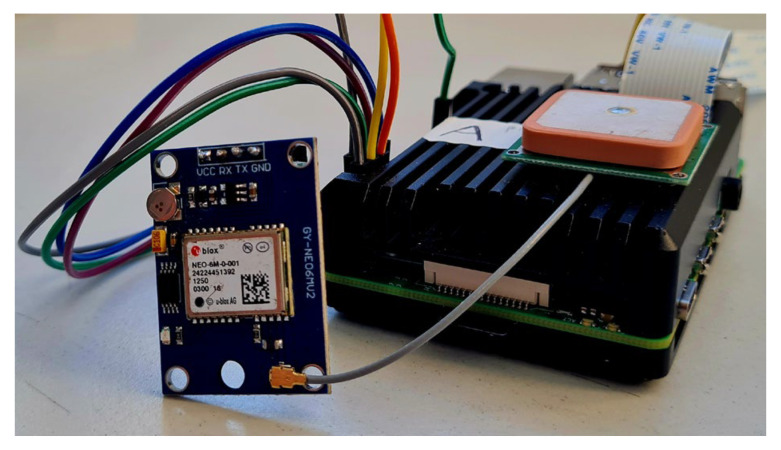
A view of the GPS antenna and the U-blox GPS module connected to Rpi 4B.

A U-blox NEO-6M GPS module (NEO-6M GPS Module, Figure 3) [49] has been connected to Rpi 4B (Table 2) to add GPS tags to images. The goal is to upload captured images of potholes into a GIS platform to enable analysis and mapping of potholes in the urban road network: this can assist the road manager in planning and prioritising road maintenance activities based on the severity and location of potholes. This procedure has been studied to define the coordinates of the potholes to repair, which are the necessary input for the TMR to reach and fix the identified potholes.

More in detail, the U-blox mini GPS module NEO-6M is a receiver that uses single-point positioning with C/A Code of L1 frequency from the GPS constellation and receives augmented data from satellite-based augmentation systems. In standard scenarios, the single-point positioning technique with single L1 frequency and civilian code has a typical horizontal position error of 13 m at a 95% probability level. Satellite-based augmentation systems (SBAS) can reduce errors and improve data integrity, availability, and continuity. The GPS module’s update rate was 1 Hz. The Python library GPSD [50] allows the acquisition of PVT data on the Raspbian environment through the NMEA protocol. The obtained GPS data includes geographic coordinates, geometric height, UTC time, and velocity.

#### 2.1.2. Camera Module

**Figure 4:**
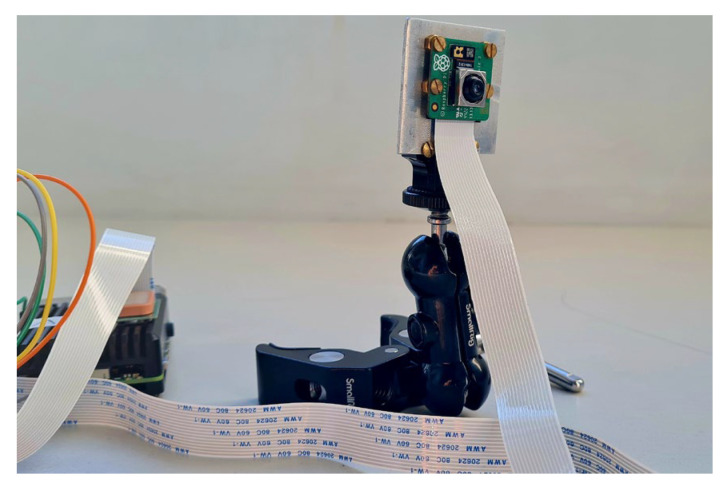
Rpi Camera Module v3 connected to Rpi 4B.

A Raspberry Pi Camera Module v3 [51] (Rpi Camera Module v3, Figure 4) allowed the collection of overlapping 2D digital photos of potholes to generate the 3D pothole model.

The Rpi Camera Module v3 features a Sony IMX708 image sensor with a high signal-to-noise ratio (SNR) and built-in 2D Dynamic Defect Pixel Correction (DPC). It also features Phase Detection Autofocus (PDAF) for rapid autofocus, supports HDR mode, and has the IR cut filter. The 25 × 24 × 11.5 mm camera has a resolution of 11.9 megapixels and a sensor size of 7.4 mm diagonally. The Camera Module v3 interfaces with the Raspberry Pi via the Camera Serial Interface (CSI) port, which enables high-speed data transfer between the camera module and the Rpi 4B.

### 2.2. Data Acquisition

**Figure 5:**
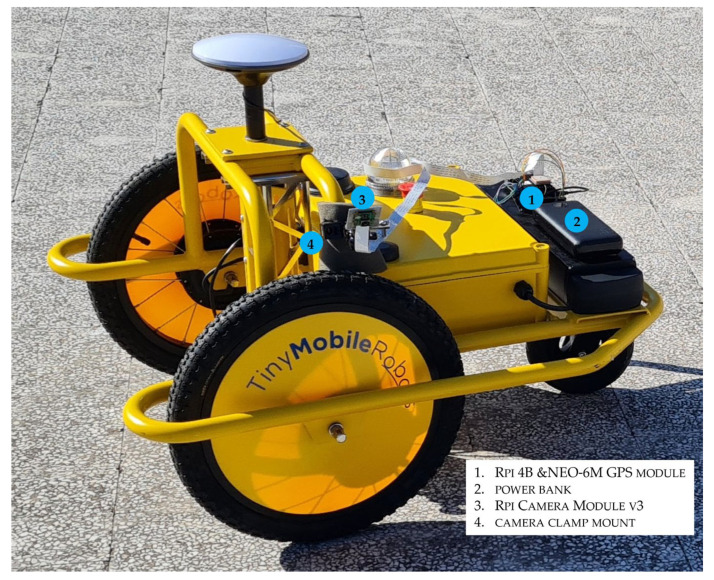
The proposed integrated system.

The hardware platform was on the small autonomous carrier (Figure 5) whose cost is not herein disclosed due to agreements signed by the InfraROB partners. The Rpi 4B and its power supply (a power bank) have been fixed in the rear part of the robot. The GPS antenna was in the upper part of the single-board computer case. A camera clamp mounts the camera on the robot chassis, and a sponge material attenuates the vibrations transmitted to the module. Preliminary test shootings returned the best position of the Rpi Camera Module v3 about height (1 m from the road surface) and view angle (45° from the vertical).

**Figure 6:**
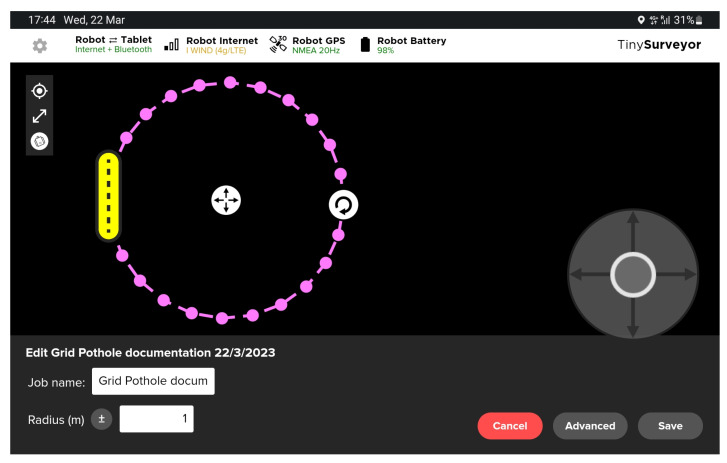
TMR path setting.

The robot has a control app that provides a custom template for the potholes mapping task. As shown in Figure 6, considering the centre of the pothole as the centre of a circle, the robot can follow a circular path all around the pothole by defining the Radius value as input. Several circles with different radii in the range of 1.0–1.4 m around the pothole need to take enough photos for a complete and detailed pothole 3D reconstruction. 

The circular path has been discretised in 30 points, describing circumferential arcs of equal length. The robot velocity driving to waypoints in automatic mode was 0.4 m/s. By pausing for 6 s at each point, the robot allows the camera capturing at least one picture of the pothole in static conditions for each point as the camera mounted on the robot automatically takes a picture per 5 s.

#### Pothole Images Acquisition Using Python Script

A Python script has been implemented in Rpi 4B to capture geotagged images using Rpi Camera Module v3 and NEO-6M GPS module. The program has an infinite loop that captures an image and adds to it GPS metadata every 5 s. Within this loop, the program obtains the GPS coordinates, captures an image using the Picamera library [52], saves it to a JPEG file format, and then adds GPS metadata to the image file using the piexif library [53]. The Picamera library is a Python interface providing easy access to the camera module of the Raspberry Pi. The library supports several features, including capturing images and video in various formats and resolutions and configuring camera settings such as brightness and exposure. The piexif library is another Python library that simplifies reading and writing metadata (EXIF, IPTC, XMP) in JPEG image files, including camera settings and geolocation data. The library also supports new image files with metadata, allowing you to add custom metadata to images.

At boot time, Rpi 4B has been connected automatically to the internet network to realise remote access and launch the command routine without being in situ. In addition, the storage has been set up automatically at the end of the survey phase in a cloud accessible from the PC equipped for post-processing.

### 2.3. Photogrammetric Data Processing

In this study, the authors exploited Bentley ContextCapture [54] to create 3D models of real-world scenes using multiple photos. However, it has to be noticed that other non-commercial and commercial specialized software (e.g., Pixpro [55], Agisoft Metashape [56], MicMac [57], Meshroom [58], VisualSFM [59]) are available to generate 3D models and shall be investigated for further comparative analyses, that are beyond the scope of this study.

Photographs around the pothole need to provide information about the geometry and appearance of the scene from different viewpoints. It is necessary to have enough overlap between the photos—the overlap between consecutive photographs should typically exceed two-thirds—so that the software can detect common features and accurately match them. 

ContextCapture uses photogrammetry techniques to analyse the photos and reconstruct the 3D geometry with aerotriangulation. In this study, user tie points gave scaling constraints during aerotriangulation. These user-tie points have been identified by selecting at least two photos with overlapping views and specifying the distance between them. After the aerotriangulation step is completed, a 3D mesh model of the scene composed of interconnected polygons represents the surveyed surface. The resulting mesh can be exported in various formats as STL: the 3D printer designed as part of InfraROB requires this format file as input. Tools are also available to perform measurements such as area and volume.

### 2.4. Computation of Pothole Fill Material

Given the pothole’s volume (*V*) and the mixture characteristics, Equation (1) gives the mixture weight to be extruded (*W*). Specifically, the bulk density of the mixture (*ρ*) obtained in a previous study is 2.12 g/cm^3^ [39].

(1) W=ρ×V

### 2.5. GIS Technologies to Map Potholes

The position of the centre of the pothole where the robot must initially go and stand is obtained by reading the GPS data acquired with the NEO-6M GPS module during the survey. Before starting, the GPS antenna coincides with the centre acquired for defining the path around the pothole. The filter on the data to identify the position when the robot stands in the centre of the pothole is possible by comparing acquisition times of GPS data and images captured by the camera, already activated during the approach to the pothole. The open-source software QGIS [60] was used to visualise the volume, the amount of fill material, and the pothole’s position as a point on the map, with an attached image of the pothole. A Python script automated this operation. The code loads the list of pothole coordinates, volumes, and backfill material from a CSV file, creates a new point layer “POTHOLES” in QGIS, and adds the referenced points, the attributes “Volume” and “Material to Fill Potholes” to the layer. It also allows the user to select the photo for each point by providing a photo path as input.

## 3. Results

### 3.1. Laboratory Tests

**Figure 7:**
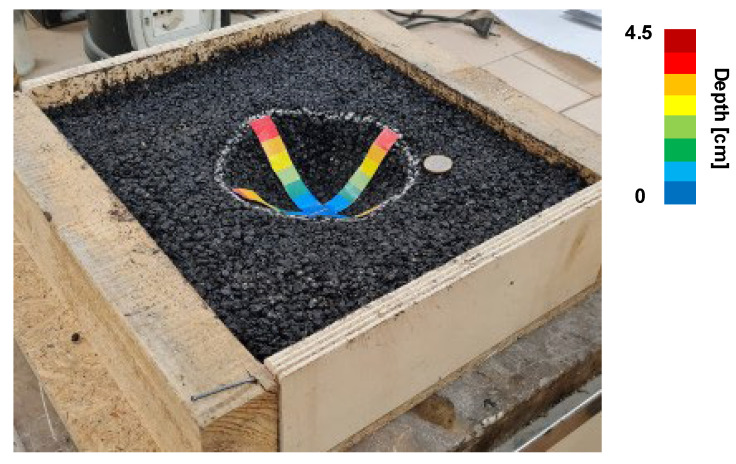
Asphalt tile simulating a real pothole.

Laboratory tests were conducted on an asphalt tile simulating a real pothole (Figure 7). A 37 × 32 × 8 cm wooden formwork was filled with cold-mix asphalt and compacted until the upper surface was horizontal. A bowl shape has been opened near the tile’s centre to simulate a pothole.

The robot collected the centre of the asphalt tile by including its coordinates from the NEO-6M GPS module. During its riding, the Rpi Camera Module v3 shot 90 static pictures (image dimensions: 4608 × 2592 pixels) within minutes. Three circular trajectories with 1.0, 1.2, and 1.4 m radii were set. The photos were transferred to a computer as dataset input to reproduce a detailed 3D model of the pothole with Bentley ContextCapture software. The desktop Lenovo ThinkStation P620 with Windows 11 Pro 64-bit, an AMD Ryzen Threadripper PRO 3955WX 16-Cores processor, 32.0 GB of RAM, and an NVIDIA Quadro RTX 5000 have been used for the photogrammetric processing. In this study, the processing time (queue) was about 25 minutes, and the quality report generated by the software revealed an average ground resolution equal to 0.40267 mm/pixel.

A scale constraint during aerotriangulation was entered by selecting in 2 different photos user tie points A and B corresponding to the ends of one of the sides of the wooden formwork of known dimensions (31.5 cm) and then specifying the distance between them. Aerotriangulation main settings are described in Table 3.

**Figure 8:**
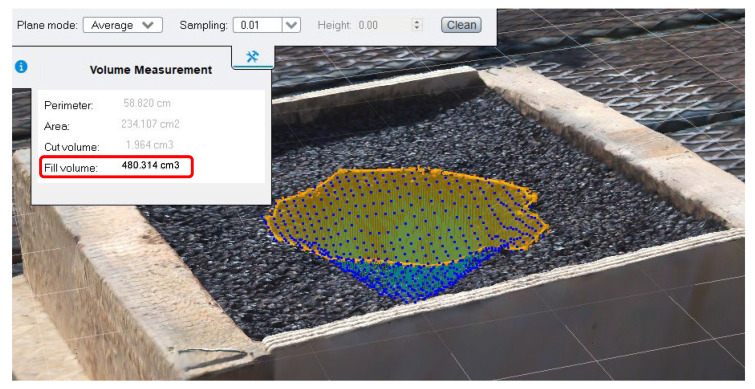
Determining pothole fill volume using photogrammetry software.

After the aerotriangulation step, the 3D model was created (software’s default processing settings) and then questioned to evaluate the volume of the pothole (Figure 8). More in detail, this software task includes manually drawing the boundaries of the pothole as a Region of Interest (ROI). Once the ROI is defined, Bentley ContextCapture calculates the volume by comparing the model surface with a reference plane (i.e., the top surface defined by its mean plane determined as a function of the pothole edge). This approach involves dividing the 3D model into a grid of prisms, which represent an approximate discretization of the volume occupied by the model. 

**Figure 9:**
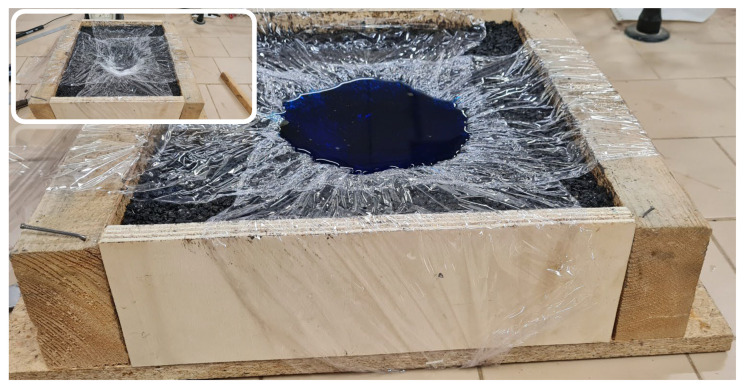
Measuring pothole fill volume in lab tests.

The actual volume was then measured in the laboratory by filling the pothole with water to verify the output from the software. For this purpose, the pothole was waterproofed by applying a synthetic thermoplastic material film; after which the distilled water contained in a graduated cylinder (±5 mL) was poured in (Figure 9).

The water volume at which water leaked out of the pothole was 485 mL: it confirmed that the proposed methodology is effective as the water volume returned by the software is very close (a relative error of 1%).

The weight of the material needed to fill the pothole was then determined (Equation (1)). In the experimental set-up, it was 1.02 kg.

**Figure 10:**
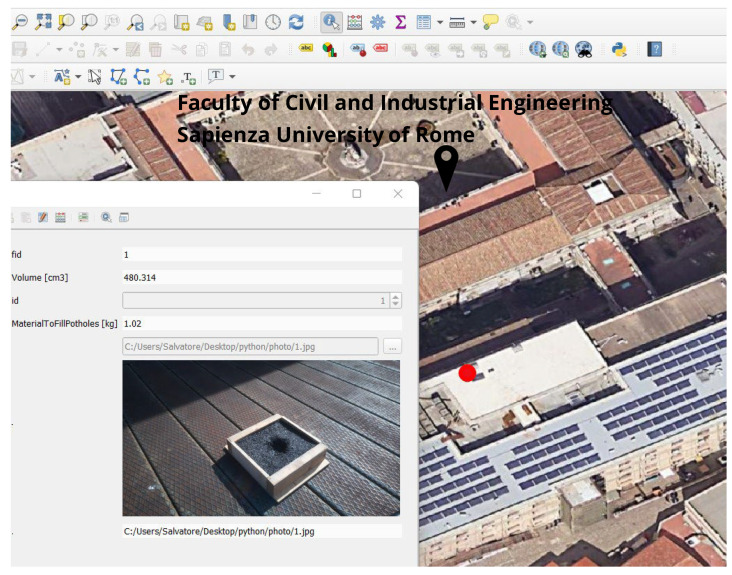
Pothole information in GIS. The red dot locates in the centre of the pothole.

Finally, all the information was uploaded and mapped in QGIS (Figure 10). 

### 3.2. On-Site Tests

**Figure 11:**
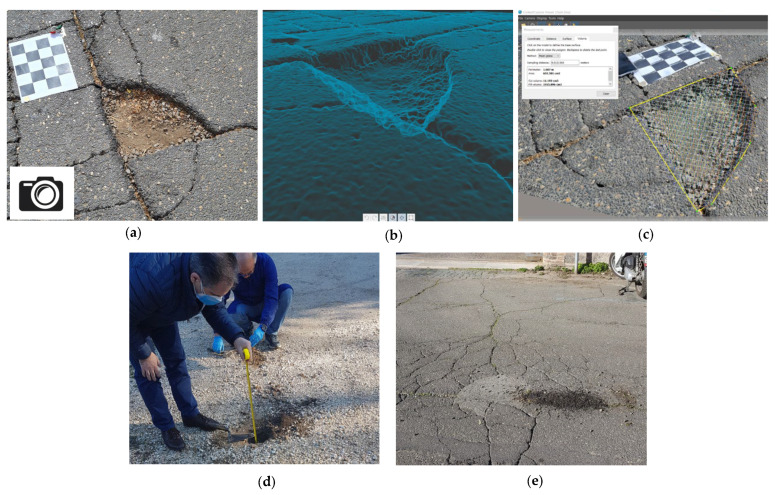
Main phases of the presented methodology. (**a**) Photos of the pothole from different points of view; (**b**) 3D mesh model of the scene; (**c**) Volume computation using software tools; (**d**) On-site inspection by qualified personnel; (**e**) Amount of filling material.

Ten on-site tests were carried out in a private parking area to validate that the system works in an uncontrolled environment and not only in the laboratory. Figure 11a–e shows how the main phases of the methodology have been applied. 

Table 4 shows the computed pothole volume and the actual amount of filling material (Equation (1)) used to fix potholes after compaction.

The results confirmed that the laboratory set-up did not differ from the actual in situ conditions, as the extruded loose material (after compaction) effectively fixed the potholes by restoring surface evenness.

## 4. Conclusions

The most frequent distress on urban asphalt pavements are cracking, deformation, bleeding, and potholes. Potholes on the wearing course and the consequent material loss significantly impact road safety since they can damage vehicles and cause unforeseen accidents for road users. The essential needs of maintaining the integrity, performance, and safety of road infrastructures have been tackled in InfraROB, an ongoing research project funded by the EU research programme Horizon 2020 that aims to promote autonomous robotized solutions and modularization. A specific project task focuses on a 3D printer able to extrude a cold asphalt mixture composed of 100% RAP and fix small potholes.

This study presents a low-cost, high-performance pothole monitoring system. A Raspberry-based prototype has been developed and mounted on an autonomous and remote-controlled robot to avoid safety hazards to workers during field surveys. The proposed integrated system involves a camera and GPS modules managed by a mini-computer Rpi 4B. The robot can describe a circular path around the pothole at 0.4 m/s. Meanwhile, the prototype collects the coordinates of the pothole centre and shoots overlapped 2D images in static conditions and from different view angles. Laboratory tests on an asphalt tile simulating a real pothole validated the proposed methodology. The collected images (i.e., 90) are then processed through photogrammetric techniques using Bentley ContextCapture software to create the pothole 3D model and calculate the volume to fill. The processing time has been fast because the entire queue last about 25 min. Nevertheless, any photogrammetry software that returns data in the format required by the 3D printer in the filling phase could be used.

The bulk density of the mixture designed (i.e., 2.12 g/cm^3^) in this InfraROB task combined with the computed volume returns the weight to be extruded. Finally, for managing the available information as the coordinates of the pothole centre, the calculated volume and backfill material, and one photo are uploaded and mapped on a GIS platform. 

The proposed approach is a valuable tool for road agencies to monitor road potholes and implement preventive maintenance strategies reducing risks for road workers and staying within budget constraints. On-site tests demonstrated that the adopted procedures can be implemented without modification in an uncontrolled environment (e.g., open-to-traffic pavements) because the laboratory set does not differ from in-situ conditions.

Further developments include integrating Building Information Modelling (BIM) and GIS can be leveraged to support the development of sustainable built environments and provide an effective PMS. This approach is becoming important according to European legislation [61] that encourages the adoption of BIM in public works and infrastructure projects. As for the on-site activities, the analysis will focus on a 3D printer to extrude the mixture and repair potholes. Finally, field tests will be performed on different damaged sections in an urban road network to monitor the behaviour of the new equipment. Future studies also include adapting the system to different distress (e.g., all type of cracks).

## Figures and Tables

**Table 1:** Severity-based pothole classification.

Maximum Depth [mm]	Average Diameter [mm]		
	100–200	200–450	450–750
13–25	L	L	M
25–50	L	M	H
>50	M	M	H

**Table 2:** Connections between the NEO-6M GPS module and the Rpi 4B.

Raspberry Pin	Sensor Pin
3V3	VCC
GND	GND
GPIO 14	RX
GPIO 15	TX

**Table 3:** Aerotriangulation main settings in Bentley ContextCapture for the study processes.

Parameters	Settings
Final rigid registration	Registration constraints
Key point density	High
Pair selection mode	Default
Component construction mode	OnePass
Blockwise colour equalization	Enabled
Splats	Enabled

**Table 4:** On-site test results.

Pothole	Pothole Volume [cm^3^]	Computed Filling Material [kg]
I	392	0.83
II	1024	2.17
III	925	1.96
IV	745	1.58
V	1519	3.22
VI	429	0.91
VII	1925	4.08
VIII	1259	2.67
IX	2057	4.36
X	415	0.88

## Data Availability

The data presented in this study are available on request from the corresponding author. The data are not publicly available due to confidentiality reasons.
